# Fabrication and Characterization of Bimetallic Silica-Based and 3D-Printed Active Colloidal Cubes

**DOI:** 10.1021/acs.langmuir.5c00815

**Published:** 2025-05-03

**Authors:** Silvana A. Caipa Cure, Daniela J. Kraft

**Affiliations:** † Department of Soft and Biological Matter, Huygens-Kamerlingh Onnes Laboratory, Leiden University, PO Box 9504, 2300 RA Leiden, The Netherlands; ‡ Department of Soft and Biological Matter, Huygens-Kamerlingh Onnes Laboratory, PO Box 9504, 2300 RA Leiden, The Netherlands

## Abstract

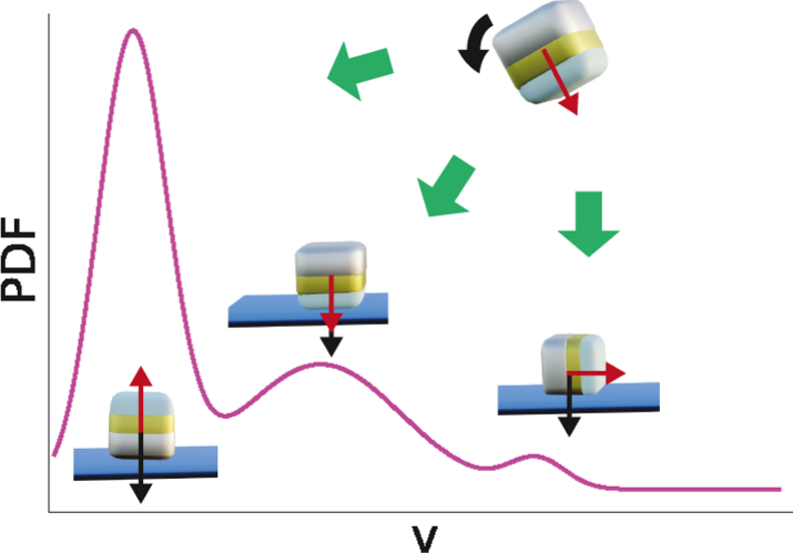

Simulations on self-propelling active cubes reveal interesting behaviors at both the individual and the collective level, emphasizing the importance of developing experimental analogues that allow testing these theoretical predictions. The majority of experimental realizations of active colloidal cubes rely on light actuation and/or magnetic fields to have a persistent active mechanism and lack material versatility. Here, we propose a system of active bimetallic cubes whose propulsion mechanism is based on a catalytic reaction and study their behavior. We realize such a system from synthetic silica cuboids and 3D-printed microcubes, followed by the deposition of gold and platinum layers on their surface. We characterize the colloids’ dynamics for different thicknesses of the gold layer at low and high hydrogen peroxide concentrations. We show that the thickness of the base gold layer has only a minor effect on the self-propulsion speed and, in addition, induces a gravitational torque during sedimentation. For low activity, this gravitational torque orients the particles such that their velocity director is pointing out of the plane, thus effectively suppressing propulsion. We find that a higher active force can remedy the effects of torque, resulting in all possible particle orientations, including one with the metal cap on the side, which is favorable for in-plane propulsion. Finally, we use 3D printing to compare our results to cubes made from a different material, size, and roundness and demonstrate that the speed scaling with increasing particle size originates from the size-dependent drag. Our experiments extend the fabrication of active cubes to different materials and propulsion mechanisms and highlight that the design of active particles with anisotropic shapes requires consideration of the interplay between shape and activity to achieve favorable sedimentation and efficient in-plane propulsion.

## Introduction

In recent years, much effort has been put into the fabrication and characterization of synthetic microswimmers that are able to move autonomously and in a controlled fashion.^1−3^ These active systems are appealing not only for their out-of-equilibrium dynamics^3−5^ but also on account of their ability to model biological and physical systems,^6,7^ biomedical cargo transportation,^8^ and environmental remediation techniques.^9^ Experimentally, a range of approaches have been explored to propel artificial microswimmers: from colloids that are activated by light or magnetic fields^10,11^ to swimmers with built-in polarity such as Janus particles that are capable of exhibiting self-electro-, thermo-, diffusio-, and chemo-phoresis.^12,13^

A significant fraction of experimental studies on self-propelled particles have focused on colloidal spheres and rods: from the early work of Howse et al. on Janus spherical particles with a catalytic platinum (Pt) patch,^12^ to the development of rod-shaped particles consisting of gold (Au) and platinum segments,^13^ to more cutting-edge fabrication techniques such as mesoporous silica spheres where the Pt is situated on the inside.^14^ In contrast, experimental research on other geometries is quite limited, although progress has been made with fabricating active anisotropic particles, for example, prepared by assembling spheres^15,16^ or based on lithography and 3D microprinting, as is the case for L-shaped,^17^ helical,^18^ tori,^19^ and crescent-shaped designs.^20^

Active colloidal cubes exhibit a rather simple form of shape anisotropy, yet a variety of interesting emergent properties have been predicted for these microswimmers. 3D simulations of active sphero-cubes in confinement showed unusual phase behavior with a second-order freezing transition and predicted new crystal structures such as the sheared cubic crystal at high densities.^21^ Moreover, simulations of hard active cubes in 2D have yielded compelling findings on the influence of shape on collision efficiency, which leads to lower critical packing fractions than those observed for active hard spheres for the onset of mobility-induced phase separation (MIPS), and the formation of multiple stable clusters during MIPS that persist for long time scales.^22^ Therefore, experimental model systems that allow testing of these theoretical predictions are considered highly valuable.

Experimentally, hematite (α-Fe_2_O_3_)-based colloidal particles are by far the most successful realization of self-propelled colloidal cubes. Although not truly cubic in shape, composite colloids made from hematite cubes encapsulated by a polymer sphere made from 3-methacryloxypropyl trimethoxysilane were shown to exhibit self-propulsion when exposed to a UV light source in a hydrogen peroxide (H_2_O_2_) medium.^2,23^ Research that followed showed that their active motion can be controlled by taking advantage of the permanent magnetic moment of this iron oxide to perform tasks such as cargo docking and transport.^24^ A more recent experimental realization of this system showed that the deposition of Pt metal on one of the sides of the hematite cubes enhances their activity in the presence of chemical fuel and when illuminated by a UV source.^25^ Despite these advancements in hematite cubic colloids, there is no realization of active cubes at the microscale that (i) does not depend on light activation to have a persistent active mechanism and (ii) consists of materials other than hematite. Light-independent persistent self-propulsion is key to probing particles’ behavior within setups in which having a UV source is not possible or not desired due to photobleaching of fluorescent dyes, sample heating, or observation of light-sensitive reactions. Additionally, moving away from hematite into more versatile materials expands the fabrication possibilities for these kinds of anisotropic particles.

In this paper, we combine experimental techniques to fabricate colloidal cubes with electrocatalytic self-propulsion mechanisms to introduce activity. For this, we use both synthetic silica cuboids templated from hematite and 3D-printed microcubes to achieve a cubic shape, followed by the deposition of gold and platinum on the cubes’ surface to implement the active mechanism. We qualitatively describe and quantify the system’s dynamics for different thicknesses of the Au layer at low and high H_2_O_2_ concentrations. Our observations show that, for a low H_2_O_2_ concentration, cuboid colloids do not undergo orientational or dynamical changes even for increasing thickness of the metallic layer on their surface. We attribute the similar propulsion speeds to gravitational torques that reorient the cuboid particles during sedimentation such that their metal caps are located at the bottom. However, as the H_2_O_2_ concentration is increased, the particles’ speeds follow a trinodal distribution, hinting at a different sedimentation behavior that leads to cuboid particles with all possible orientations. We also study the effect of material, size, and shape on the cubes’ self-propulsion speeds by comparing the synthetic particles to 3D-printed ones and demonstrate that decreasing speeds with increasing particle size originate from an increased drag. Our experimental technique provides an alternative route to fabricate active cubes from versatile materials such as silica and polymers, expanding the fabrication possibilities beyond those currently available, removing the need for light activation, and allowing for experimental validation of theoretical predictions at high particle densities.

## Experimental Section

### Materials

H_2_O_2_ (35%, in water) and propylene glycol monomethyl ether acrylate (PGMEA, >99.5%) were purchased from Sigma-Aldrich. 2-Propanol (IPA,9, 99%) was purchased from VWR Chemicals. All materials were used as received, unless stated otherwise. All solutions were prepared from deionized water with 18.2 MΩ cm resistivity using a Millipore Filtration System (Milli-Q Gradient A10).

### Preparation of Silica Cuboids

Colloidal cuboids with edge-to-edge length 2.0 ± 0.3 μm were previously synthesized for the work of Shelke et al.^26^ Briefly, monodisperse pseudocubic hematite particles (edge-to-edge length 1.52 ± 0.05 μm) were prepared from condensed ferric hydroxide gel^27^ and then coated with a silica layer by a Stöber procedure.^28^ After dissolution of the hematite cores with HCl, hollow silica shells with cuboid geometry were obtained. The shape of these cuboids lies between a sphere and a cube and can be described by , where *L* is the face-to-face length and *m* is a shape parameter typically ranging between 2.5 < *m* < 4^29^ and *L* = 2.0 ± 0.3 μm.

### Particle Printing

Cubes with sharp edges and corners were fabricated using a commercially available two-photon polymerization (2PP) 3D microprinter (Photonic Professional Gt, Nanoscribe GmbH) equipped with a 63× oil-immersion objective (Zeiss, NA = 1.4) in dip mode. Conditional on the resolution of the equipment, the cubes were printed with a side length of 4 μm. First, the cubes were designed and rendered using Autodesk Inventor and Describe. Then, the structures were printed onto a fused silica substrate using the commercial photoresist IP-Dip purchased from Nanoscribe GmbH.^18^ After printing, the particles were developed by 30 min of submersion in PGMEA and a 2 min dip in IPA and subsequently left to dry. Once developed, the particles were ready for sputter-coating.

### Metallic-Layer Deposition

Cuboid silica particles and 3D-printed particles were sputter-coated with thin metallic layers to integrate a propulsion mechanism. For this, 50 μL of diluted colloidal dispersion was deposited onto a silica substrate (2.5 cm × 2.5 cm) via spin-coating using an SCS 6800 Spin Coater series at 1983 rpm for 30 s. The concentration was tuned manually such that spin-coating resulted in a monolayer. This step was not necessary for the 3D-printed particles because they were already distributed in a monolayer on top of a silica substrate.

**Figure 1:**
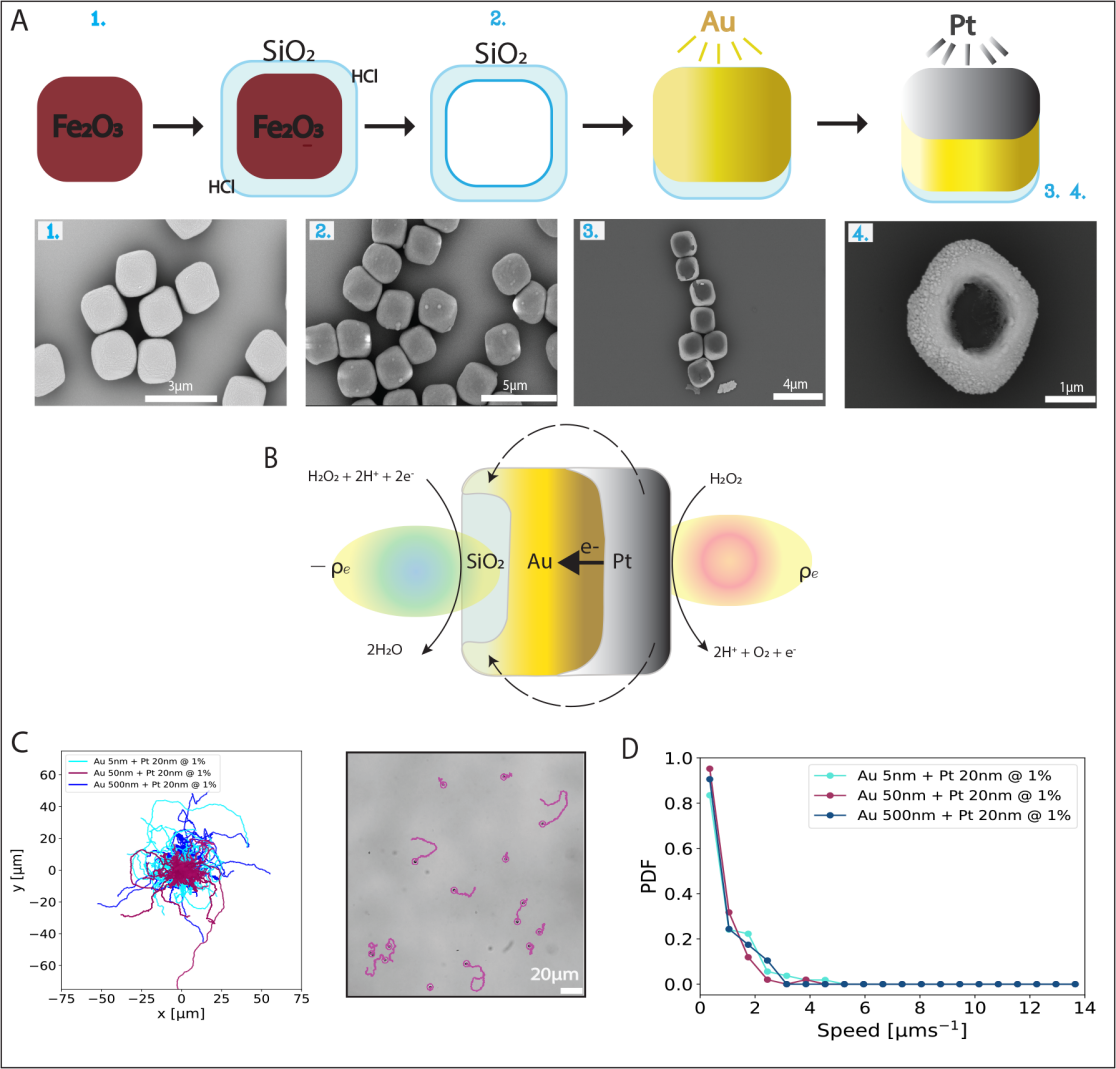
(A) Schematic of the fabrication process for silica-based bimetallic cuboids, accompanied by SEM images of (A1) hematite cuboids, (A2) hematite cuboids coated with silica and hollow silica cuboids after acid dissolution of the hematite core, (A3) metal-coated silica cuboids with a 5 nm Au base layer followed by a 20 nm Pt top layer, and (A4) 500 nm Au base layer followed by a 20 nm Pt top layer. (B) Schematic of self-electrophoresis of a bimetallic Au–Pt silica cuboid adapted from Moran, 2017.^32^ The H_2_O_2_ is oxidized on the platinum’s (cathode) surface driving an electron flow into the gold (anode) layer and a proton flow on the fluid. This reaction leads to an asymmetric charge density distribution that generates electric fields and a slip flow of the fluid around the particle that propels the particle in the opposite direction. (C) Trajectories centered at the origin, recorded over the course of 30 s at 20 fps for active cuboids coated with different thicknesses of a Au layer followed by a 20 nm Pt layer, in the presence of H_2_O_2_ 1%v/v. Accompanied by a sample snapshot of 30 s trajectories depicted on top of the corresponding bright-field image. (D) Mean speed distributions of >30 particles for each of the layers’ thicknesses, in a H_2_O_2_ 1%v/v solution. The speeds were fitted after calculating the MSDs from trajectories recorded via optical microscopy and fitting them to ⟨*r*^2^⟩ = 4*D*_0_δ*t* + 2*v*^2^δ*t*^2^ with δ*t*_max_ = 1 s.

The substrate containing the monolayer of particles was then sputter-coated by using the following vacuum systems consecutively. We used a high-vacuum Leybold-Heraeus Z400 sputter-coater with custom modifications to obtain a gold coating of all exposed surfaces of the cubes. In this system, the target is close to the substrate, and sputtering is performed at ≈10^–5^ mbar (Ar @ 54 sccm, Direct Current Eectrode Positive: 1 kV), yielding a robust layer that covers most of the particle. This results in cuboids that have five of the six faces covered by a gold layer. We next employed a low-vacuum (10^–2^ mbar) Cressington 208HR sputter-coater to create a thin metallic cap of platinum for coating only the top of the particles. The Pt target was purchased from Micro to Nano (Ø57 × 0.2 mm, 99.99%). The consecutive sputtering with gold and platinum in the two systems yielded particles that had five out of six faces covered by a gold layer, and on the top half was a platinum layer. Different metal layer thicknesses were tested: 20–500 nm of Au (in high vacuum) and 5–20 nm of Pt (in low vacuum). A schematic of this procedure can be found in Figure 1A. Sputter-coated particles were then recovered from the substrate via sonication and redispersed in water.

### Particle Observation

To characterize the surface of the particles after sputter-coating, scanning electron microscopy (SEM) images were taken with a Thermo-Fisher Apreo SEM. For this, particles were deposited onto a silicon SEM substrate and allowed to dry in air before imaging.

Information on the dynamics of the metal-coated particles was extracted from bright-field microscopy observations in a custom-made microscope holder in the presence of 1 or 5%v/v H_2_O_2_ in water. A fresh H_2_O_2_ solution was made for every measurement day to avoid depletion of the fuel due to light or temperature. Particles were imaged using a Nikon Eclipse Ti microscope with 60× water immersion (NA = 0.7) and 20× dry (NA = 0.5) objectives. Movies of 30–120 s were taken for all samples at 20 frames per second (fps), unless stated otherwise. The surface area fraction of particles was ϕ_particles_ = 0.01–0.05% in relation to the total area of the field of view. Samples were kept for only a maximum of 30 min to avoid H_2_O_2_ depletion, convection effects due to bubble formation, and to prevent an increasing number of particles from sticking to the substrate.

### Data Analysis

The videos were analyzed using the Crocker–Grier algorithm implemented for Python as Trackpy^30^ to extract *XY* trajectories of the particles. Although Trackpy is intended to track particles with round features, at the magnifications used, the features could be approximated to circles, yielding reliable tracking results. From the trajectories identified with the algorithm, mean square displacements (MSDs) were calculated and fitted to ⟨*r*^2^⟩ = 4*D*_0_δ*t* + *v*^2^δ*t*^2^, where *D*_0_ is the translational diffusion coefficient, *v* is the particle speed, and δ*t*_max_ = 1 s is the maximum lag time for the fit, which is lower than the rotational diffusion time of the particles.^4,12^ The fit allowed us to estimate the particles’ speed (*v*) and their translational diffusion coefficient (*D*_0_). For all samples, >30 particles were analyzed per sample to get statistically relevant information, with the exception of printed particles for which sometimes it was possible to obtain only *n* ≈ 10, given the low particle density that the 3D microprinting methods yield. Particles that were stuck to the substrate, i.e., not displaying activity or Brownian motion, were not taken into account during the analysis.

## Results and Discussion

### Dynamics of Bimetallic Au–Pt Silica-Based Cuboids with Increasing Metallic-Layer Thickness

Our strategy for the fabrication of active cubes consisted of using a combination of two metals on the surface of cuboid silica particles to promote both self-diffusio- and electrophoretic propulsion in a H_2_O_2_ solution. For this, hematite colloids (Figure 1A1) were used to template silica cuboids with edge-to-edge length 2.0 ± 0.3 μm (Figure 1A2) via a Stöber procedure. The cuboids were spin-coated onto a glass slide and sputter-coated in a high-vacuum system from above to cover five out of six faces of the particle’s surface with a 5 nm Au layer. We then employed a low-vacuum sputtering system that allowed us to deposit a 20 nm thick Pt layer on the top half of the cuboids; see Figure 1 A3,A4. The corresponding SEM images of each step are shown below the schematic. This technique allowed us to introduce an anodic layer (Au) to promote self-electrophoresis, to the well-established self-propulsion mechanism in which a Pt layer drives the asymmetric decomposition of H_2_O_2_.^31^ Self-electrophoretic activity results from the interaction between a self-generated electric field and a charged colloidal surface. For Au–Pt bimetallic swimmers in a H_2_O_2_ solution, a proton concentration gradient as a result of redox reactions establishes an electrical dipole around the particle that, when coupled with the free charges present in the particle’s electrical double layer, induces an electroosmotic slip around the particle, causing it to swim^32−34^; see Figure 1B. Additionally, at low fuel concentrations, self-electrophoretic swimmers have proven to propel significantly faster than swimmers driven by other active mechanisms,^4,32,35,36^ which can be advantageous for studies at higher particle densities, such as was shown by Klongvessa et al.^35^ High fuel efficiency at relatively low fuel concentration avoids disturbance of the experiments by bubble formation while still allowing for high particle velocity.

In contact with 1%v/v H_2_O_2_, bimetallic silica cuboids with 5 nm Pt and 20 nm Au layers displayed self-propulsion. Their dynamics were observed by bright-field microscopy, and their position was tracked using Trackpy, as shown in Figure 1C, light blue. The speeds were extracted from their short-time mean-squared displacement, and a probability density function of the speed was obtained from >30 particle trajectories as shown in Figure 1D, light blue. The mean particle speed extracted from the particles’ short-time mean-squared displacement (*n* > 30) was 0.94 ± 0.85 μm s^–1^. The uncertainty corresponds to 1 standard deviation and shows the broad distribution of the data.

We followed by studying the effect of the thickness of the gold layer on the swimming speed of bimetallic Au–Pt silica-based cuboid particles. The reasons for that were 2-fold. First, we were inspired by the high speeds exhibited by Pt-coated Au spheres reported by Klongvessa et al. and Theurkauff et al*.,* where particles attained a speed of up to three body lengths per second at low H_2_O_2_ concentrations.^3,35^ Second, the effect of the platinum’s thickness has already been investigated for electrophoretic systems of bimetallic particles, where a directly proportional relation between the catalyst’s thickness and the particles’ speed was observed.^25^

For the Au base layer, two other thicknesses were assessed (50 and 500 nm), while the top Pt layer was kept at a constant thickness of 20 nm. We found that all cuboid particles possess similar speed distributions regardless of the thickness of the metallic base layer, as can be observed by the overlap in Figure 1D. In line with this, the arithmetic mean speeds of 0.94 ± 0.85, 0.71 ± 0.59, and 0.76 ± 0.64 μm s^–1^ for 5, 50, and 500 nm Au layer thickness, respectively, agreed within the error. In addition, all mean speeds were less than half a body length per second and thus significantly smaller than those found by Klongvessa et al. and Theurkauff et al*.,*^3,35^ suggesting that thick layers of gold are not able to recreate the fast propulsion speed properties seen in Pt-coated solid Au particles.^35^ We conclude that at 1%v/v H_2_O_2_ the thickness of the gold layer does not have an effect on the translational speed of the cuboids.

### Dynamics of Bimetallic Au–Pt Silica-Based Cuboids with Increasing Metallic-Layer Thickness at a Higher Fuel Concentration

**Figure 2:**
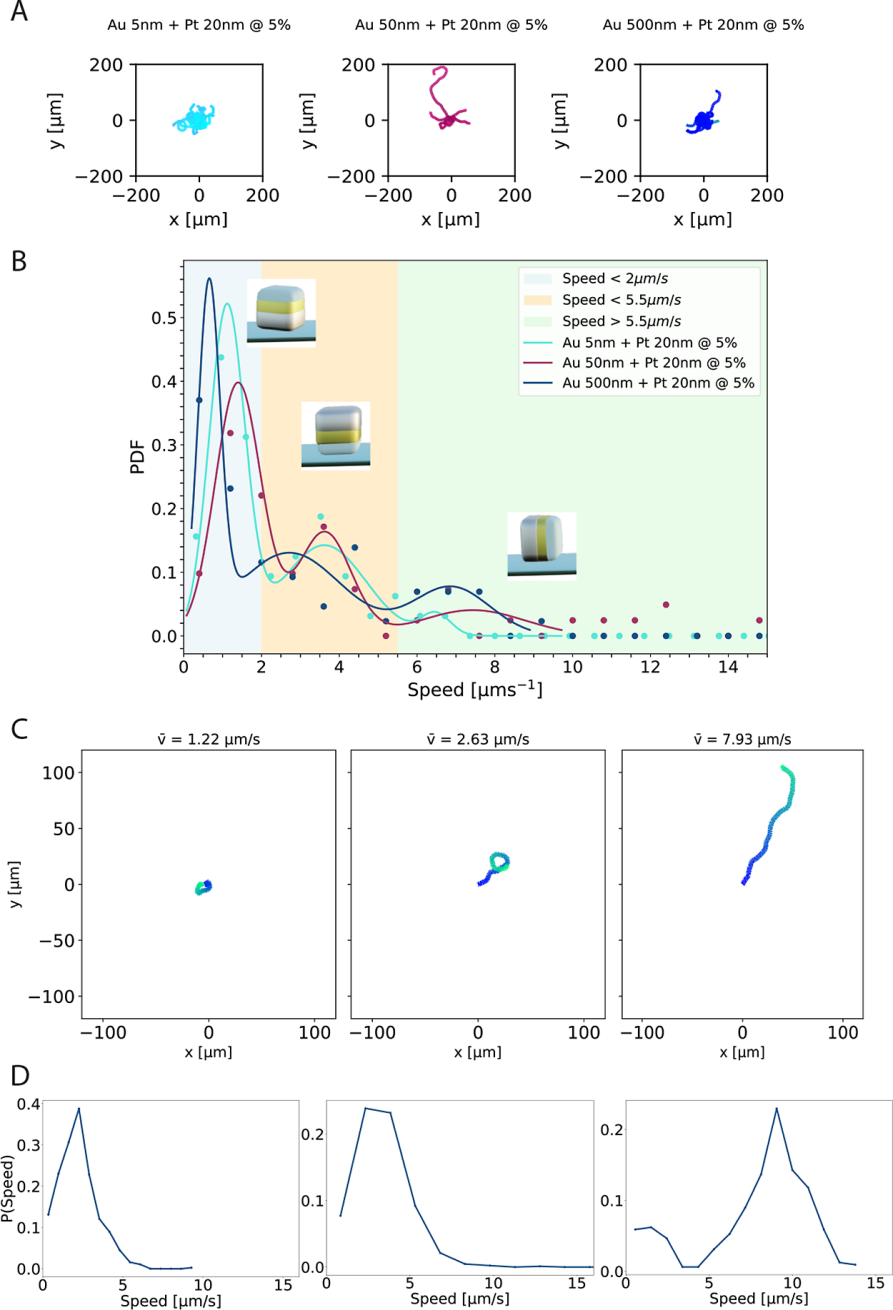
(A) Trajectories centered at the origin, recorded over the course of 30 s at 20 fps for active cuboids coated with a 5, a 50, and a 500 nm Au layer, followed by a 20 nm Pt layer, in the presence of H_2_O_2_ 5%v/v. (B) Mean speed distribution of >30 particles for each of the layer thickness configurations, in a H_2_O_2_ 5%v/v solution. For each gold layer thickness, we performed a three-component Gaussian fit as follows: . Here, *A*_1_, *A*_2_, and *A*_3_ are the amplitudes; μ_1_, μ_2_, and μ_3_ are the means; and σ_1_, σ_2_, and σ_3_ the standard deviations. Colored areas indicate the three populations as identified by the fits. (C) Exemplary trajectories centered at the origin, for active cuboids coated with a 500 nm Au layer followed by a 20 nm Pt layer taken from each of the three populations found in (B), in the presence of H_2_O_2_ 5%v/v. Particle speed increases from left to right. These were recorded over the course of 30 s at 20 fps. (D) Instant speed distributions corresponding to the trajectories shown in (C); speeds were obtained by averaging over 2 s.

With the aim of making our system more active, i.e., higher mean speeds, and gaining a better understanding of the active mechanism, we performed the same measurements as for the previous section, but this time for 5%v/v instead of 1%v/v H_2_O_2_, as shown in Figure 2. Increasing the fuel concentration up to 10%v/v in H_2_O_2_–Pt catalytic systems is a reliable way of obtaining higher colloidal speeds.^12,34^

Figure 2B depicts the measured speed distributions for the colloidal systems probed at 5%v/v H_2_O_2_, as extracted by quantitative particle tracking from bright-field microscopy videos. The distributions are obtained from >30 particle trajectories, and the velocity is extracted from their short-time mean-squared displacement. Compared to the data for 1%v/v H_2_O_2_, the mean speeds of the particles were significantly higher, i.e., 2.24 ± 1.61, 4.00 ± 3.88, and 2.75 ± 2.42 μm s^–1^ for a 5, 50, and 500 nm thick Au layer, respectively, which represent an average speed increase of 3.8× upon fuel addition. Again, the uncertainty corresponds to one standard deviation and shows the broad distribution of the data. The resulting speed distributions were fitted to a three-component Gaussian fit as follows: , where *A*_1_, *A*_2_, and *A*_3_ are the amplitudes; μ_1_, μ_2_, and μ_3_ are the means; and σ_1_, σ_2_, and σ_3_ are the standard deviations. Here, we excluded data above 10 μm s^–1^ to obtain a higher fit quality of the significant measurements. The resulting peaks allowed us to divide the result into three populations: a significant fraction of particles showed speeds below 2 μm s^–1^ for all Au thicknesses, an intermediate fraction showed speeds in between 2 and 5.5 μm s^–1^, and some of the particles coated with the 50 and 500 nm Au possessed speeds above 5.5 μm s^–1^. This behavior differs quite a lot from what was observed at a lower fuel concentration and hints at an influence of the Au layer thickness on the particles’ speed as well as at possible orientational differences. We will return to this in the next section.

Finally, from the distributions in Figure 2B as well as from the sample trajectories in Figure 2A, one can notice that at 5%v/v H_2_O_2_ some particles attain velocities higher than 10 μm s^–1^ and reach up to 15 μm s^–1^. The speeds of these fast cuboids are comparable to those found in the literature for magnetic and photoactivated hematite colloids with similar cap thicknesses.^25^ We note that at a higher (8%v/v) concentration of H_2_O_2_, the formation of bubbles interfered with data collection.

We further tested whether individual particles taken from the different populations show differences in their motion and speed distributions. We show the trajectory and corresponding speed distribution for a representative particle taken from one of the three populations in Figure 2C,D. Speeds were calculated by averaging over 2 s to reduce noise. Slower particles show very short-range trajectories without preferred directionality, while faster particles have longer and more persistent trajectories. In addition, while each particle possesses a distribution of speeds, as is also the case for spherical Janus particles,^37^ they are clearly different from the speed distribution of the ensemble (Figure 2B) and distinctly different from particles stemming from other populations.

### Sedimentation Induced Orientation and Its Effect on Self-Propulsion

The results from the previous sections reveal a clear difference for our cuboid system between low and high fuel concentrations: at low fuel levels, the average particle speed remains unchanged regardless of the increasing thickness of the Au layer; however, as the fuel level increases, three distinct populations emerge in the speed distribution, with two of them exhibiting significant speeds. These observations suggest that besides self-electrophoresis, other effects are dominating the particles’ dynamics.

An anisotropic particle shape such as the cubic shape of our particles implies the possibility for different orientations of the particles and hence their directors with respect to the surface. For particles made from materials with densities that are high compared with that of the surrounding solvent, thermal fluctuations can be insufficient to induce rotations of the particle after sedimentation. The orientation of the particle with respect to the surface during sedimentation then can become an important factor to consider, as particles with their director oriented out of the plane of the substrate will not be able to propel. We hypothesize that this is the origin of the different speed distributions seen at different H_2_O_2_ concentrations.

The bimetallic coating induces a pronounced density difference throughout the particles’ surface, which affects their sedimentation. The silica cuboids are hollow, but Pt and Au are both on average 10 times denser than silica. Differences in the thickness of the metal coating therefore are likely to influence the orientation of the cap with respect to the substrate during sedimentation. A similar torque has previously been observed experimentally for spheres with a pronounced mass anisotropy, which preferred to orient with the metallic cap toward the substrate when sedimenting in the absence of fuel,^38−40^ and shown to lead to orientational quenching close to a substrate.^41^ Indeed, Figure 1A3,A4 shows the SEM micrograph of two different dried samples of bimetallic silica cuboids after being left to sediment in water. Given the thickness and density of the metallic layers, the region corresponding to the silica base points upward, as signified by the dark circle at the center of the cubes.

**Figure 3:**
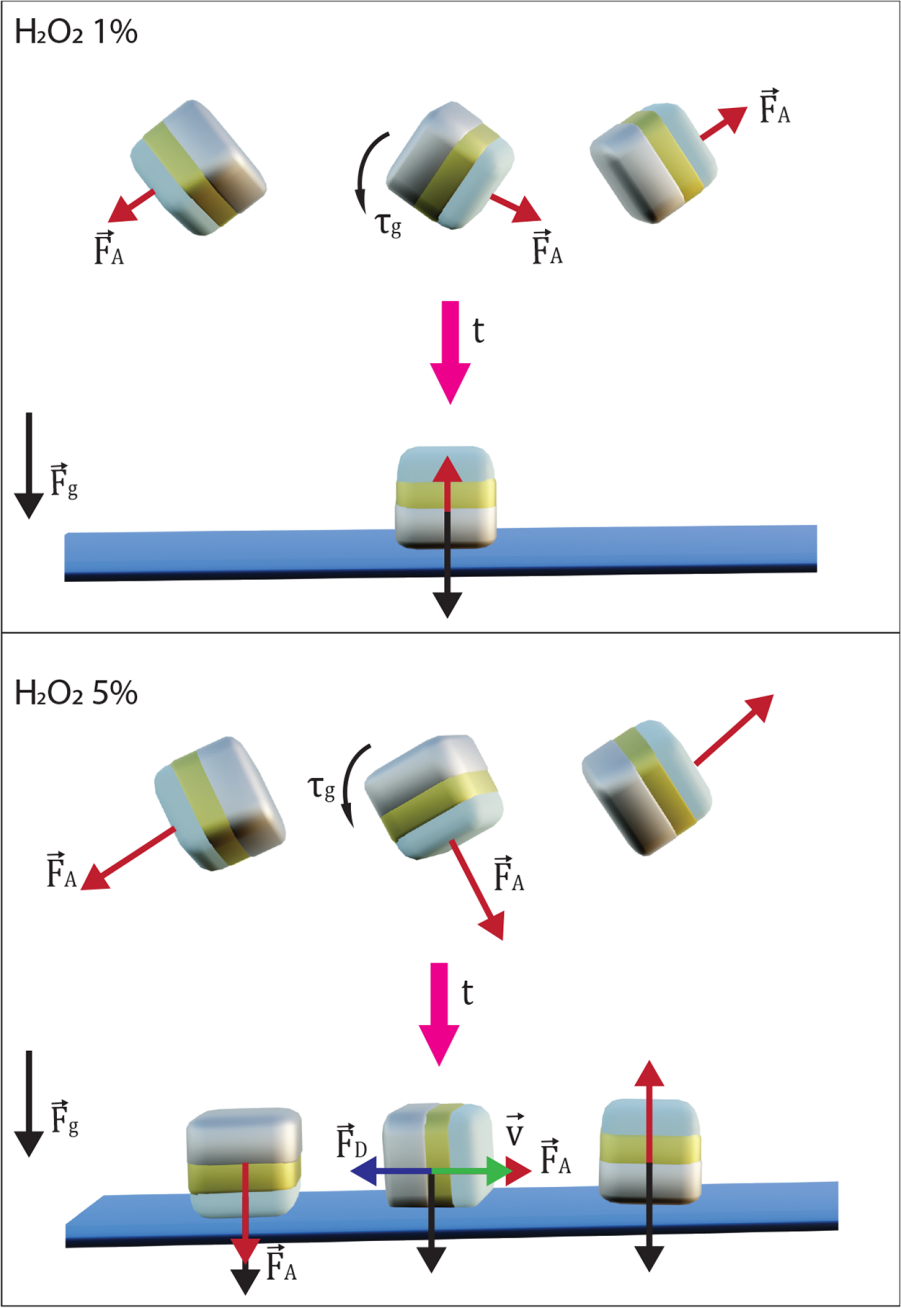
Activity affects sedimentation, which leads to different orientations of the particles once they reach the surface. Top: at low activity, the cuboids are reoriented by the gravitational torque τ_g_ such that they land with their metal cap at the bottom on the substrate. This results in the active force  pointing out of the plane and thus a low net speed . Bottom: at high activities, activity can enhance/slow down the sedimentation speed depending on the initial particle orientation, leaving too little/sufficient time for the gravitational torque to reorient the particles with their metal cap down. As a result, all possible orientations are found. Once the particles move, the drag force  is equal and opposite to the active force.

The low velocities of the particles observed in the presence of 1%v/v H_2_O_2_ suggest that the mass anisotropy induces torques and thus cap-down orientations during sedimentation, even when the particles self-propel. Once the active cuboid particles have reached the substrate metal-side downward, their orientation with respect to the plane normal of the substrate plane must remain locked. Unlike many Janus particles that also show strong orientation quenching of the director with respect to the surface due to a balance between the chemical activity-induced torque and the mass anisotropy-induced torque,^42−46^ the orientation of the cuboids is locked due to a combination of gravity and their anisotropic shape: once sedimented onto one side, rotation requires work against gravity. Their high density difference with respect to the solvent density leads to small gravitational heights and thus effective confinement. The active force , which points upward out of the substrate’s plane, apparently is not able to induce rotations either, possibly due to hydrodynamic flows that usually lead to orientation quenching; see Figure 3, top. Low velocities could also stem from the scenario in which the  vector points into the plane but are less likely given the high mass anisotropy.

In contrast, at 5%v/v H_2_O_2_, there are three distinct speed populations of particles. We hypothesize that these three populations stem from the three different orientations the active cubes can adopt with respect to the substrate: metal cap on the bottom, metal cap on the top, and metal cap on the side. The higher activity seems to make it possible for the cubes to reach all three orientations instead of mainly one. To rationalize this observation, we consider that the active particles originally have random particle orientations. The direction of the active force  is coupled to that orientation and may point toward or away from the bottom substrate, i.e., away from or along the gravitational force vector . If the active force has contributions in the direction of the gravitational force, sedimentation speed will be enhanced; otherwise, it will be slowed down. At the same time, the mass anisotropy introduces a gravitational torque τ_g_, which tends to align the particle with its metal side toward the bottom. However, the torque-induced rotation takes some time, during which the particle might reach the substrate, and it does so the faster, the larger the contribution of the activity vector along the direction of the gravitational force. If the activity vector had antiparallel contributions to the gravitational force, sedimentation is slowed down and the particle has more time to rotate due to its mass anisotropy, which will slow down sedimentation further, until it ultimately lands with the metal side down on the substrate (Figure 3, bottom). At low values of the active force, the gravitational torque almost always has sufficient time to align the particle with the metal cap down, as sedimentation speed is never strongly enhanced by the activity.

We attribute the population with the highest speeds of up to 15 μm s^–1^ to cuboids with a metal coating on the side. In this orientation, their active force vector is oriented parallel to the plane and the particles are most likely able to fully harness the chemically induced activity for their self-propulsion. These speeds, which are on the order of two to seven particle sizes per second, are also in line with refs (3 and 35) that inspired our particle design.

The population with the lowest particle speeds below 2 μm s^–1^ shows similar speeds as the particles at 1%v/v H_2_O_2_, suggesting that it corresponds to particles oriented with their metal cap down. While we would not a priori expect particles oriented with their metal cap up to have a faster speed than those with a cap down, the decreasing number of particles with increasing speed suggests that the orientation with the metal side up or on the side must be less easily attainable. We thus propose that the population with the lowest speed corresponds to particles with their metal cap down, while the population with intermediate speeds corresponds to particles with their metal cap on the top. The speed difference between the metal cap down and metal cap up orientations may stem from different strengths of osmotic flow contributions induced on the substrate.^43,47,48^ We finally note that despite the increased activity, the particles were never observed to leave the bottom glass substrate independent of their orientation. This suggests that the density of the cubes and of the metallic layers is significantly higher than that of the surrounding fluid such that the active force is not able to overcome the combination of gravitational force and fluid flow induced affinities once sedimented.

Thus, while two metals are needed for self-electrophoresis, the thickness of the base Au layer has only a minor effect on the self-propulsion speed. The overall thickness of the metals matters, since it determines the mass anisotropy and thus gravitational torques. However, we find that the magnitude of the active force affects the sedimentation behavior of bimetallic silica-based cuboids and partially counteracts the effect of the gravity-induced torques. A higher activity allows for a higher fraction of cubes to land in orientations where the active force is pointing into the substrate or parallel to the substrate and thus propels faster, yielding three speed populations.

### Dynamics of Bimetallic Au–Pt 3D-Printed Cubes

**Figure 4:**
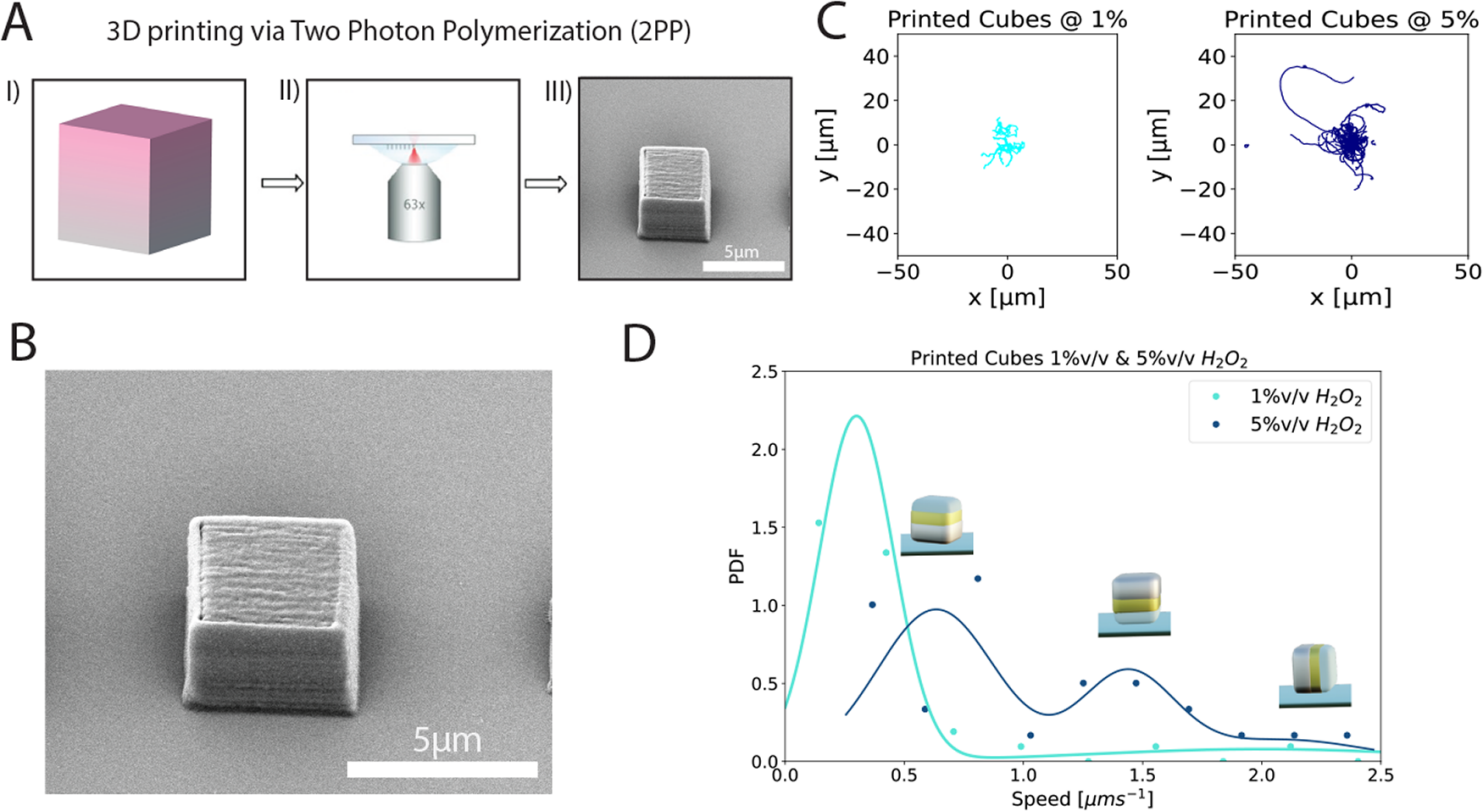
(A) Schematic of the printing procedure: (I) design of the Computer Assisted Design (CAD) model, (II) printing using the Nanoscribe (III) development of the print to obtain the final shape. Adapted from Doherty et al.^18^ B) SEM micrograph of 3D-printed cubes (4 μm side length). (C) Trajectories centered at the origin, recorded over the course of 30 s at 20 fps, in the presence of 1 and 5%v/v H_2_O_2_, for active printed cubes coated with a 10 nm Au base layer followed by a 10 nm Pt top layer. (D) Mean speed distributions of printed cubes coated with a 10 nm Au base layer followed by a 10 nm Pt top layer, in the presence of 1 and 5%v/v H_2_O_2_. Speeds were obtained from fitting MSD = 4*D*_0_δ*t* + 2*v*^2^δ*t*^2^ with δ*t*_max_ = 1 s to the trajectories recorded via optical microscopy. For 5%v/v H_2_O_2_, the Gaussian fit was performed using , where *A*_1_, *A*_2_, and *A*_3_ are the amplitudes; μ_1_, μ_2_, and μ_3_ are the means; and σ_1_, σ_2_, and σ_3_ the standard deviations.

To test the influence of size and definition of the cubic shape as well as extend the technique to other materials, we apply the same bimetallic coating onto 3D-printed particles with a cubic shape. 3D printing via two-photon lithography allows tuning the shape parameter of the cuboid such that it becomes a perfect cube with sharp edges and corners (*m* ≫ 1). We design cubes with a CAD program and print them using a 2PP-based mechanism (Nanoscribe Photonic Professional Gt), as depicted in Figure 4A. The resulting particle is shown in Figure 4B and has a side length of 4 μm.

As before, we coated the cubes with a thin bimetallic layer consisting of a 10 nm Au layer at the base and a 10 nm Pt layer on top. We observed the motion of these bimetallic 3D-printed cubes using bright-field microscopy, at 1 and 5%v/v H_2_O_2_ and analyzed their trajectories using TrackPy. Sample trajectories and speed distributions can be found in Figure 4C,D. For the active bimetallic 3D-printed cubes, we can observe three speed populations similar to what has previously been seen for the active bimetallic silica-based cuboids under the same conditions: at 1%v/v H_2_O_2_, most of the cubes sediment in an orientation that yields no or low net displacement. However, at 5%v/v H_2_O_2_, the stronger active force again leads to faster particles, with more frequently an orientation that allows in-plane self-propulsion. Therefore, under similar conditions, there is no significant difference between the active mechanism for 3D-printed cubes with sharp edges and synthetic cuboids with rounded edges, despite the variation in size and material. For both fabrication techniques, self-propulsion is dominated by the sedimentation behavior, which leads to locked particle orientations on the substrate due to a combination of shape and gravitational confinement.

The main difference between the 3D-printed system and the silica-based system is the overall lower speed: the peaks in the speed distribution for the 3D-printed cubes at a high fuel concentration are located at 0.62, 1.44, and 2.09 μm s^–1^, respectively, and the average speed of the system is 1.03 ± 0.58 μm s^–1^, which is considerably lower than for the synthetic cuboids (refer to Figure 2C). We rationalize this lower value by considering that the drag force experienced by a cubic particle is size-dependent. In a uniform flow field, the drag force is defined as , where ξ_cube_^T^ is the translational friction coefficient, which was found to scale with the side length *L* as ξ_cube_^T^ = 1.384 × 6πη*L* in simulations performed by Okada et al.^49^ Assuming that the active force is the same and given that the side length of 3D-printed cubes is *L*_3D_ = 4 μm and *L*_Si_ = 2.0 ± 0.3 μm for the silica cuboids, we expect their speeds to relate as *v*_3D_/*v*_Si_ = *L*_Si_/*L*_3D_ = (2.0 ± 0.3)/4 = 0.50 ± 0.08. This agrees very well with the ratio of the measured mean speeds of 1.03 ± 0.58 μm s^–1^ (observed for the 3D-printed cubes) and 2.24 ± 1.61 μm s^–1^ (for 5 nm Au-coated cuboids), which corresponds to 0.46 ± 0.42. Thus, we can attribute the different speeds mainly to the size-dependent drag. Other effects, such as drag differences due to more rounded or sharp edges, intrinsic hydrodynamics of the active system that cannot be described using the active/drag force alone, effects originating from the presence of the surface such as slip, charge effects, and roughness,^43^ or differences in the extent of the interface between the two metals seem to play a minor role.

## Conclusions

In this work, we investigated the 2D dynamics of active bimetallic Au–Pt, silica-based cuboids, and 3D-printed cubes. These electrocatalytic colloids are active through self-diffusio- and electrophoresis. Unlike the majority of the existing experimental systems, our cubes do not rely on magnetic or UV actuation to have a persistent self-propulsion mechanism and can be fabricated from materials such as silica and polymers. The colloids were studied via bright-field microscopy, allowing us to qualitatively describe and quantify their dynamics for different thicknesses of the Au layer at different fuel concentrations.

For bimetallic silica-based cuboids at 1%v/v H_2_O_2_, only a small fraction of the cubes exhibited significant activity, while the majority showed little to no net displacement. We argued that this behavior is related to the metal-side downward orientation that the particles adopt during and maintain after sedimentation on the substrate, given the thickness and density of the metallic layers, and the shape-dependent quenching of the particles’ orientation once they reach the substrate.

At higher fuel concentrations of 5%v/v H_2_O_2_, three populations appeared in the speed distribution for all thicknesses probed: a population with a similar, low speed as for low fuel concentrations, which we hypothesize stems from particles oriented with their metal cap down, and two faster populations, which we believe to originate from particles with their metal cap oriented to the top and to the side. We hypothesized that the higher active force either enhances or reduces the sedimentation speed, which increases or decreases, respectively, the probability for a particle orientation that is favorable for in-plane propulsion.

3D-printed cubes were studied to understand the effect of material, shape, and size on the dynamics of cubic colloidal systems. The mechanism for active bimetallic printed cubes proved to be similar to that of their silica analogues, where the in-plane behavior is determined by the sedimentation behavior, rather than self-electrophoresis, which can be tuned with different activity levels. We attributed the lower self-propulsion speeds of the 3D-printed particles compared to those of the silica-based cuboids to the increased drag due to their larger size.

Our experimental realization of bimetallic self-electrophoretic active cubes does not rely on magnetic or light activation for persistent self-propulsion, which is important for setups in which having a UV source is not possible or not desired. The bimetallic coating approach can be straightforwardly applied to other particle shapes.

However, in line with an earlier work on spheres, we find that metal coatings induce torques during sedimentation due to the inherent mass anisotropy. This, together with the anisotropic shape, can lead to a significant fraction of particles with low or no net speed. Even in the presence of a significant activity, we find that an anisotropic particle shape will lead to different orientations with respect to the substrate and thus different propulsion speeds. Therefore, when designing active particles, especially when intending to obtain homogeneously active samples, for example, for experiments at high particle density, one thus should take this into consideration, for instance, by circumventing shape-induced orientational locking by designing shapes that can rotate away from the mass-anisotropy-induced orientation on the substrate or by tuning the density of the surrounding fluid to counteract the gravitational effects.^39^
